# Genicular artery embolization for knee osteoarthritis: A systematic review of sham-controlled randomized trials

**DOI:** 10.1016/j.jor.2025.07.022

**Published:** 2025-07-25

**Authors:** Fathi Milhem, Muhammad Takhman, Mohamed S. Elgendy, Anas Abu Zahra, Sarah Saife, Sakeena Saife, Waseem Shehadeh, Mohammad Bdair, Omar Abu-Khazneh, Yazan Hamdan, Qutayba Z. Ayaseh, Orabi Hajjeh, Ayesha Younas, Walaa Abu Alya, Ahmad Mohammad, Anwar Zaitoun

**Affiliations:** a Department of Medicine, Faculty of Medicine and Health Sciences, An-Najah National University, Nablus, Palestine; b Faculty of Medicine, Tanta University, Tanta, Egypt; c Allama Iqbal Medical College, Lahore, Pakistan; d Cleveland Clinic Mercy Hospital, Cleveland, OH, USA; e Department of Internal Medicine, Hurley Medical Center, Flint, MI, USA

## Abstract

**Background:**

Knee osteoarthritis (KOA) is a degenerative joint disease associated with chronic pain and functional decline. Genicular artery embolization (GAE) is a minimally invasive intervention that targets abnormal synovial neovascularization. This systematic review evaluates the efficacy and safety of GAE compared with sham procedures in patients with symptomatic KOA.

**Methods:**

A systematic search of PubMed, EMBASE, Scopus, and Web of Science was conducted through March 2025. Only sham-controlled randomized controlled trials (RCTs) enrolling adults with symptomatic KOA were included. Data were extracted on study design, patient characteristics, interventions, outcomes, and adverse events. Risk of bias was assessed using the Cochrane RoB 2.0 tool.

**Results:**

Three sham-controlled RCTs with a total of 138 patients were included. GAE demonstrated short-term pain reduction, particularly in VAS scores, with one trial showing a significant improvement at 1 month (−50.8 vs −0.5). KOOS pain scores improved modestly across studies but lacked statistical significance. Functional outcomes were mixed; one trial reported significant improvement in WOMAC function, and another found enhanced quality of life in patients undergoing complete embolization. No serious adverse events occurred; minor complications such as catheter-site bruising were infrequent and self-limited.

**Conclusions:**

GAE appears to be a safe and minimally invasive treatment that may provide short-term symptomatic relief in select KOA patients. However, limited sample sizes, methodological variability, and short follow-up periods constrain definitive conclusions. Larger, standardized trials with longer follow-up are necessary to confirm efficacy and optimize patient selection.

## 1 Introduction

Osteoarthritis (OA) is a prevalent and debilitating degenerative joint disease characterized by progressive cartilage degeneration leading to pain, stiffness, and functional impairment. It's one of the most common causes of chronic pain and disability worldwide, particularly affecting the knee joint.^1^ Knee osteoarthritis (OA) poses a significant burden, contributing to reduced mobility, diminished quality of life, and increased healthcare costs.^2^

**Fig. 1:**
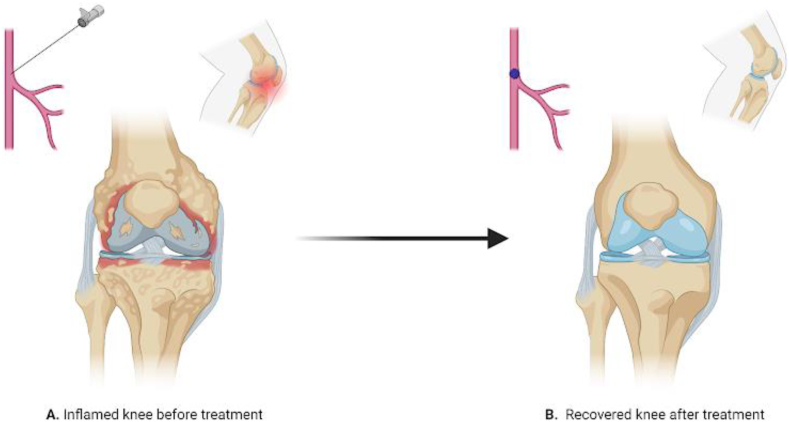
The impact of full embolization on knee pain relief. (A) highlights persistent inflammation and pain before the procedure. (B) The post-embolization state, where reduced blood flow to pain-mediating structures leads to significant pain alleviation.

Traditional management strategies for KOA include a stepwise approach of lifestyle modifications and pharmacological interventions, including non-steroidal anti-inflammatory drugs (NSAIDs), intra-articular injections, and in severe cases, surgical options such as total knee arthroplasty (TKA).^3^ Despite this, variable efficacy is up to 30 % of patients who underwent TKA surgery are dissatisfied due to unmet presurgical expectations, particularly regarding functional improvement, potential side effects, and invasiveness of surgical procedures, highlighting the need for alternative treatment modalities.^4^ In recent years, genicular artery embolization (GAE) emerged as a promising, minimally invasive treatment option for patients with symptomatic KOA, particularly those who are ineligible for or wish to avoid surgery.^5^ This endovascular technique selectively targets the abnormal neovascularization and associated inflammation in the synovium, which are the key contributors to OA-related pain. By selectively embolizing the genicular arteries, this procedure aims to reduce the blood flow to the hypervascular synovium, thereby alleviating pain and improving joint function.^3^ (Fig. 1).

This systematic review aims to evaluate the current evidence on GAE for KOA, assessing its efficacy, safety profile, and comparative effectiveness against other treatment modalities since early research and clinical trials demonstrated encouraging outcomes in terms of pain reduction and quality of life enhancement in both the short and long term.^3^^,^^6^ Although GAE has shown some promise as a minimally invasive alternative, the available evidence is fragmented and lacks a comprehensive synthesis regarding its efficacy, safety, and comparative effectiveness. While recent meta-analyses have explored the overall effectiveness of GAE, isolating its true clinical significance is needed, which is the focus of the present review.^7^ This is the first systematic review that aims to evaluate and consolidate current evidence on the use of GAE for KOA compared to the Sham procedure, assessing its therapeutic efficacy and safety profile. The goal is to determine GAE's role in KOA management and identify areas requiring further research.

## 2 Methodology

### 2.1 Protocol registration

We conducted your meta-analysis in light of the PRISMA^1^ (Preferred Reporting Items for Systematic Reviews and Meta-Analyses) guidelines. We also followed the Cochrane Handbook for Systematic Reviews of Interventions.^2^ Our review protocol was prospectively registered in the PROSPERO (Registration ID: CRD420251053126).

### 2.2 Data sources and search strategy

Two independent reviewers conducted a comprehensive literature search across four major databases—PubMed, Scopus, Web of Science, and EMBASE—to identify relevant studies published up to March 10, 2025. The search strategy combined terms related to osteoarthritis and vascular intervention, specifically: (“osteoarthritis” OR “degenerative joint disease”) AND (“arteries” OR “artery” OR “arterial” OR “vascular”) AND (“embolization” OR “embolotherapy”). To ensure we didn't miss any important studies, we also manually reviewed the reference lists of all included articles and relevant reviews to find any additional eligible publications.

### 2.3 Eligibility criteria

We included clinical trials that were published in peer-reviewed journals and met the following PICOS criteria.

1. **Population (P)**: adult patients (≥18 years) diagnosed with symptomatic knee osteoarthritis for at least 3 months.
2. **Intervention (I)**: minimally invasive GAE.
3. **Comparison (C)**: a sham procedure by undergoing a similar but non-therapeutic intervention, which typically involves mimicking the steps of GAE.
4. **Outcomes (O)**: our primary outcomes were mean change in pain scores (the Knee injury and Osteoarthritis Outcome Score (KOOS) and Visual Analog Scale (VAS)).
5. **Study design (S):** randomized controlled trials (RCTs) only.

We did not use any limits on sex, ethnicity, or year of publication. We did not include preclinical and animal studies, non-randomized and quasi-experimental trials, studies that were observational (including cohort and case-control study designs), and reviews, books, theses, conference abstracts, protocols, letters, case reports, and case series. Trials with unclear or missing data were also excluded.

### 2.4 Study selection

For the selection of studies, we used the online tool Rayyan to facilitate screening and automatically remove duplicates. Titles and abstracts of the remaining records were independently assessed by two reviewers for potential relevance. Full-text articles that met the inclusion criteria were subsequently evaluated based on predefined PICOS (Population, Intervention, Comparison, Outcomes, and Study design) categories. Any disagreements between reviewers were resolved through consultation with a third reviewer.

### 2.5 Data extraction

Two reviewers independently extracted the data using a pre-made Excel sheet (Microsoft, USA). The sheets of extraction were divided into three domains.

(i) **Study characteristics:** (study ID, study design, registration number, geographic location, total number of participants, study arms [intervention and comparator], crossover allowance, inclusion criteria [age, severity, KL grade], primary outcome(s), maximum follow-up duration, and key conclusions).
(ii) **Baseline characteristics of the included patients:** ().
(iii) **Study outcomes:** (primary outcomes were VAS and KOOS pain scores at various time points, and Secondary outcomes included the WOMAC function subscale and KOOS subdomains related to daily living, sports, and recreation. Quality of life was evaluated using the KOOS-QOL subscale and EQ-5D-5L questionnaire, where available, in addition to Likert scales. Safety outcomes were consistently reported, with attention to both serious and minor adverse events (AEs).

Crossover periods in the included trials were excluded from the analysis following the point of crossover.

### 2.6 Quality assessment

To assess the quality of the included RCTs, we employed the updated Cochrane Risk of Bias 2 (RoB 2) tool. This framework allowed us to systematically evaluate potential sources of bias in each study. Based on the criteria outlined in RoB 2, we classified studies into one of three categories**:** “low risk,” “some concerns,” or “high risk” of bias. Each study was independently reviewed by at least two authors to ensure objectivity and consistency. In cases of disagreement, reviewers discussed their assessments to reach a consensus.

### 2.7 Outcomes assessment

The primary endpoint of this systematic review was to assess the efficacy and safety of GAE in adults with knee osteoarthritis. The secondary endpoints included the evaluation of procedural details, baseline clinical assessments, and the incidence of adverse events. Due to diverse study designs and varying populations, meta-analysis was not performed, and inter-study heterogeneity and statistical significance were not quantitatively evaluated in this qualitative synthesis.

## 3 Results

### 3.1 Study selection

**Fig. 2:**
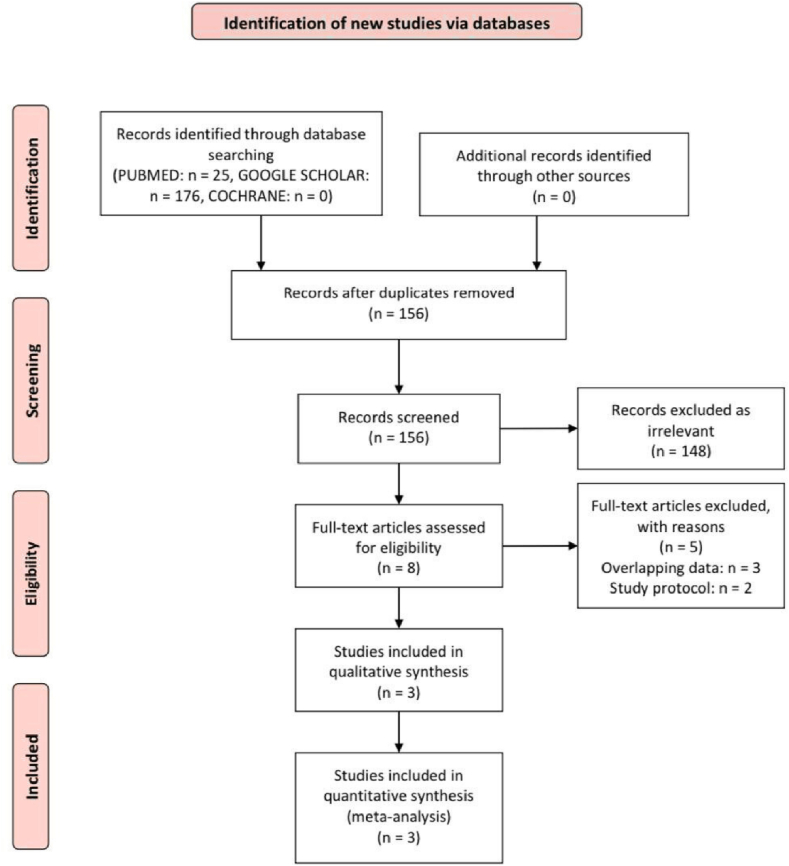
PRISMA flow chart of the screening process.

A total of 201 records were initially identified through systematic database searches. After the removal of duplicates by EndNote, 156 unique records remained for screening. Upon screening the titles and abstracts, 148 studies were excluded for being irrelevant to the inclusion criteria. The remaining eight full-text articles were assessed for eligibility. Of these, five were excluded after full-text review, then three studies met the eligibility criteria and were included. A detailed overview of the study selection process is depicted in the PRISMA flow diagram (Fig. 2).

### 3.2 Study characteristics

Three RCTs encompassing a total of 138 patients were included in this review, each investigating the effectiveness of genicular or transcatheter arterial embolization for symptomatic knee OA. Despite differences in design and methodology, several consistencies emerged. All studies targeted patients with mild-to-moderate KOA, defined as Kellgren–Lawrence grades 1 to 3 in two studies and grade 2 only in one. The embolic agents varied: Embozene microspheres were used in one study, imipenem/cilastatin sodium in another, and absorbable particles in the third. The degree of blinding ranged from single-to triple-blind, and only Bagla et al. permitted crossover to active treatment at one month for non-responders. Follow-up durations ranged from four to twelve months. Detailed summary characteristics of the included trials are shown in Table 1.

**Table 1:** Summary characteristics of included trials.

Study ID	Study design	Register number	Location	Total	Study arms		Cross-over	Inclusion criteria			Primary outcome (s)	MFUP	Conclusion
					Intervention	Comparator		Age	Severity	KL Grade			
**Van Zadelhoff et al. 2024**	Single-center, double-blinded, RCT	NCT03884049	Netherlands	58	GAE: Embozene microspheres (75/100 μm)	Sham procedure (catheter maneuvering simulated)	Not allowed	≥18 y	Mild-to-moderate	1–3	KOOS Pain scale	4 months	We did not establish a clinical effect of GAE in patients with mild-to-moderate Knee OA as GAE produced a similar effect on pain reduction as a sham GAE procedure.
**Landers et al. 2023**	Single-center, triple-blinded, RCT	ACTRN12616001184460	Australia	59	TAE: Imipenem/cilastatin sodium in contrast	Sham procedure (mimicking angiography using video)	Not allowed	18 to 75 y	Early-stage symptomatic	2 only	KOOS Pain scale	12 months	TAE might produce benefits above placebo, but only when complete embolization of all genicular arteries is performed. Further comparative studies are required before definitive conclusions regarding the effectiveness of TAE can be made.
**Bagla et al. 2022**	Multi-center, single-blinded, RCT	NCT03362957	USA	21	GAE: Embolization using 100–300 μm absorbable particles	Sham angiography	To GAE after 1 month if no advance	>40 y	Mild to moderate	1–3	WOMAC & VAS scores	12 months	In patients with mild to moderate knee OA, GAE results in symptomatic improvement greater than the sham procedure with clinically significant reduction in pain and disability.

***RCT*** randomized controlled trials, ***KL*** Kellgren–Lawrence, ***GAE*** genicular artery embolization; ***TAE*** transcatheter arterial embolization; ***Y*** year; ***KOOS*** Knee injury and Osteoarthritis Outcome Score, ***WOMAC*** Western Ontario and McMaster Universities Osteoarthritis Index, ***VAS*** visual analog scale, ***MFUP*** maximum follow-up duration, ***OA*** osteoarthritis.

The mean age across the included studies was 60 ± 8.2 years, and approximately 65.2 % of participants were female. The mean KOOS pain score at baseline was 43.9 ± 15.5, and the mean VAS pain score was 61.8 ± 20.4, indicating that participants had moderate to severe pain before treatment. Detailed patients’ characteristics are shown in Table 2.

**Table 2:** Baseline characteristics of included patients.

Study ID	Groups	Total	Age, y (SD)	Female sex, no. (%)	BMI, kg/m2 (SD)	Laterality, no. (%)		Duration of OA, y (SD)	KOOS pain (SD)	VAS pain (SD)
						Left	Right			
**Van Zadelhoff et al. 2024**	**GAE**	29	59.0 ± 8.6	19 (65.5)	29.0 ± 4.6	14 (48.3)	15 (51.7)	7.8 ± 7.4	44.4 ± 15.6	56.5 ± 17.5
	**Sham**	29	57.3 ± 8.0	16 (55.2)	31.0 ± 4.8	16 (55.2)	13 (44.8)	8.2 ± 8.3	42.3 ± 16.5	53.5 ± 19.9
**Landers et al. 2023**	**TAE**	29	61.1 ± 8.0	18 (62.1)	32 ± 7.8	NM	NM	2.5 ± 3.1	43.5 ± 17.3	NM
	**Sham**	30	60.1 ± 7.7	19 (63.3)	33.1 ± 5.3	NM	NM	1.7 ± 1.56	45.4 ± 13	NM
**Bagla et al. 2022**	**GAE**	14	63.9 ± 8.37	12 (86)	30.8 ± 8.14	9 (64)	5 (36)	NM	NM	81.3 ± 12
	**Sham**	7	62.9 ± 7.13	6 (43)	33.4 ± 10.5	1^14^	6 (86)	NM	NM	78.9 ± 10

Data are presented in mean ± SD or proportions as (%). ***SD*** standard deviation, ***No*** number, ***GAE*** genicular artery embolization, ***TAE*** transcatheter arterial embolization, ***Y*** year, ***OA*** osteoarthritis, ***KOOS*** Knee injury and Osteoarthritis Outcome Score, ***VAS*** visual analog scale, ***NM*** not mentioned.

### 3.3 Risk of bias assessment

**Fig. 3:**
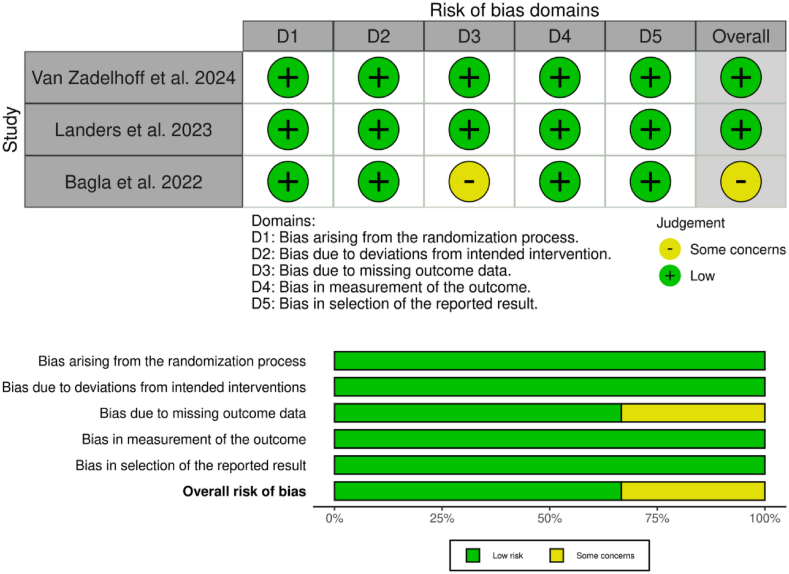
Overview of the risk of bias of the included randomized controlled trials.

All three included studies were assessed using the RoB 2.0 tool. Most risk of bias domains were rated as low risk, particularly those related to the randomization process, adherence to intended interventions, measurement of outcomes, and selective reporting. However, Bagla et al. (2022) were judged to have some concerns due to missing outcome data during long-term follow-up, which may affect the overall risk rating for the study. A summary of the quality assessment is presented in Fig. 3.

### 3.4 Outcomes

#### 3.4.1 Pain outcomes

The studies showed varying outcomes for reduction in pain after GAE. Three RCTs evaluated pain reduction using KOOS and/or VAS scores at various time points. In *van Zadelhoff* et al. Trial, the GAE group showed greater improvement in KOOS pain scores at both 1 month (18.5 vs 17.6) and 4 months (21.4 vs 18.4) compared to sham, although the differences were not statistically significant (p = 0.31). VAS scores also favored GAE at 4 months, with a greater mean reduction (−22.9 vs −13.8; p = 0.17).^8^ In *Landers* et al., KOOS pain improvements were observed at 6 months (17.6 vs 27.3) and 12 months (24.1 vs 22.2), with numerically better outcomes for GAE, though no p-values were reported.^9^ *Bagla* et al. reported a substantial reduction in VAS pain at 1 month in the GAE group (−50.8 vs −0.5; p < 0.01), although KOOS scores were not available.^10^ Overall, the results suggest potential benefits of GAE in reducing knee pain, particularly in short-term VAS outcomes, though between-group differences in KOOS scores were generally modest (Table 3).

**Table 3:** Summary of pain outcomes (KOOS and VAS) in included trials of genicular artery embolization.

Study ID	Follow-up	KOOS		VAS	
		GAE	Sham	GAE	Sham
**Van Zadelhoff et al. 2024**	1 months	18.5 (15.67)	17.570 (21.316)	N/A	N/A
	4 months	21.4 (19.59)	18.4 (17.75)	−22.90 (26.75)	−13.83 (21.07)
**Landers et al. 2023**	6 months	17.6 (22.55)	27.3 (28.131)	N/A	N/A
	12 months	24.1 (24.4)	22.2 (28.131)	N/A	N/A
**Bagla et al. 2022**	1 month	N/A	N/A	−50.8 (6.437)	−0.5 (3.504)

Values are presented as mean (standard deviation). ***KOOS*** Knee injury and Osteoarthritis Outcome Score, ***VAS*** Visual Analog Scale for pain, ***GAE*** Genicular Artery Embolization, ***Sham*** Control group receiving sham embolization, ***N/A*** Not Available.

#### 3.4.2 Functional and quality of life outcomes

In addition to pain reduction, functional improvements and quality of life measures were assessed among the included studies. The WOMAC function subscale increased by 30 points (p < 0.05) in the GAE group, according to the Bagla et al. study, indicating significant functional improvements. The Zadelhoff et al. study, on the other hand, found no significant differences between the GAE and sham groups on the KOOS subscales for quality of life, sports, or daily living, suggesting that embolization did not provide any further advantages over a placebo. Remarkably, a subgroup of patients (n = 17) who underwent complete embolization of all genicular arteries exhibited noticeably higher gains in KOOS Sports and Quality of Life scores than the control group (p = 0.012), according to the Landers et al. study (Table 2). This implies that the extent of embolization can influence how successful the procedure is, with more comprehensive embolization potentially producing better functional results.

#### 3.4.3 Adverse events

Serious adverse effects after GAE were not recorded in any of the included studies. Only five mild adverse events were reported in the sham group, but 23 were reported in the GAE group, according to the Zadelhoff et al. study (Table 2). These adverse events were generally mild and did not require medical intervention. Similarly, the Landers et al. trial found no significant complications. The most frequent adverse event was moderate bruising at the site of catheter placement, which occurred in five participants. Similarly to this, the Landers et al. trial found no significant complications. The most frequent adverse event was moderate bruising at the site of catheter placement, which happened in five participants. The safety profile of GAE appears favorable, though minor procedural-related complications may occur.

## 4 Discussion

The efficacy and safety of GAE for knee OA were evaluated in this systematic review using three randomized controlled trials with different embolization agents, varying follow-up intervals, and the use of sham controls to reduce potential bias.

A recent meta-analysis showed that GAE is generally effective, which was done by combining results from a wide variety of studies. While their work offers useful insights into GAE's potential in clinical practice, it includes both randomized and non-randomized studies and does not focus specifically on sham-controlled trials. In comparison, our review was limited to sham-controlled randomized trials, which gives a more precise picture of GAE's effectiveness by comparing with placebo effects. This difference is important, especially since procedures like GAE can have a strong placebo response, and highlights why results from studies without a control group should be interpreted carefully.^7^

In terms of pain relief, the results of the studies varied. In the study by Bagla et al. (2022), short-term improvement was observed, with significant pain reduction (KOOS Pain subscale improvement of 21.4 points) at four months in the GAE group compared to the sham group. However, this effect significantly decreased at the 12-month follow-up, suggesting that GAE may provide only short-term symptomatic relief without long-term improvement. In contrast, the study by Zadelhoff et al. revealed minimal differences between the GAE and sham groups at four months, with only a small 3.0-point difference in KOOS pain scores that was not statistically significant (p = 0.31). This finding indicates that placebo responses or other nonspecific factors may contribute to the perceived efficacy of the intervention. Landers et al. also reported pain improvement, with 41.3 % improvement in the GAE group and 29.4 % in the sham group at twelve months; however, the difference between groups was not statistically significant, making the therapeutic effect of GAE difficult to interpret conclusively.

The reviewed studies varied significantly with respect to demographic characteristics of participants, including mean age, sex distribution, BMI, baseline pain severity, and intervention modalities, such as embolization agents and methods. These differences likely impact the comparability and generalizability of the evidence. Despite such heterogeneity, the overall impression from the available evidence is that GAE holds potential for notable short-term symptom improvement in knee OA, though the duration and consistency of this benefit remain uncertain. This conclusion aligns with findings from studies on traditional treatment options such as total knee replacement (TKR), which also demonstrate short-term pain reduction. For example, a study by Nishimoto et al..^11^ reported KOOS pain subscale improvements of 19.71 and 25.54 points at four and six months post-TKR, respectively.^12^ Another study showed a significant reduction in postoperative pain using the VAS score, from 64.2 preoperatively to 19 points at 12 months postoperatively in patients undergoing TKR.^13^

One of the intriguing aspects of this review was the variation in the scope of the procedure and embolization materials. Agents ranged from Embozene microspheres to absorbable particles and imipenem/cilastatin sodium (IPM-CS). Landers et al. notably found that participants with greater completeness of embolization experienced significantly better improvements in sports performance and quality of life compared to controls. This finding suggests that achieving more complete embolization may be critical to maximizing functional outcomes.

In terms of safety, GAE demonstrated a generally favorable profile across all included studies. Consistent with findings from a systematic review and meta-analysis by Taslakian et al.,^13^ our review showed that adverse events were mostly mild and procedure-related, such as moderate bruising at the catheter insertion site. No serious adverse events or major complications were reported, supporting the notion that GAE is relatively safe compared to other interventions like TKR. In contrast, a study conducted in Australia highlighted complications associated with TKR—including infections, pulmonary embolism (PE), deep venous thrombosis (DVT), and prolonged recovery times, which occur at moderate rates and should be considered when making treatment decisions.^14^ Nevertheless, continuous monitoring and systematic reporting of adverse events remain essential to fully understand and inform safe clinical practice.

This systematic review has several notable strengths. It is the first to focus exclusively on sham-controlled randomized trials evaluating GAE for knee OA, thus providing a more robust assessment of true treatment efficacy by minimizing placebo effects. All included studies employed randomization and blinding, with one trial using a triple-blind design, enhancing internal validity. The review adhered to a pre-registered protocol and followed PRISMA guidelines, using the Cochrane RoB 2.0 tool for risk of bias assessment.

However, several limitations should be acknowledged. The total number of patients across all studies was relatively small (n = 138), limiting the ability to perform meta-analysis or draw definitive conclusions. There was considerable clinical heterogeneity in embolic agents used, outcome measures, and completeness of the procedure. One trial (Bagla et al., 2022) allowed early crossover and included a small, demographically imbalanced sample, which may have inflated short-term benefit estimates. Additionally, follow-up durations ranged from 1 to 12 months, leaving long-term effects largely unknown. Inconsistent statistical reporting across the studies further limited quantitative synthesis.

The findings of Bagla et al. (2022) warrant cautious interpretation due to methodological limitations. It had the smallest sample size (n = 21), a 2:1 group allocation, and early crossover, which could have exaggerated treatment effects. Moreover, participants had higher baseline pain (VAS: 81.3 mm), were older (mean age: 63.9 years), and predominantly female (86 %), introducing demographic imbalances not present in other trials. Thus, although short-term benefits were observed, the generalizability of the results is limited.

Based on these findings, further high-quality studies are needed. Larger-scale, multicenter trials with standardized intervention protocols, rigorous controls, and longer follow-up periods are required to better assess the long-term efficacy, duration of benefit, and safety profile of GAE for knee OA. Future research should also explore patient selection criteria based on symptom severity, radiographic stage, and demographic characteristics to optimize therapeutic decisions. Additionally, determining the most effective embolization techniques and agents through further studies is essential for improving patient outcomes and establishing evidence-based clinical guidelines for GAE in knee OA management.

## 5 Conclusion

In summary, three RCTs offer evidence that GAE can yield short-term pain and functional improvement for osteoarthritis knee patients. A single study reported significant pain relief at one month but had no statistically significant differences from sham procedures in the other studies. More complete embolization of all genicular arteries may maximize outcomes. With few reported adverse events, GAE should prove safe and minimally invasive for those seeking options beyond the standard treatments. Larger studies with longer follow-up and standardized protocols are required to confirm long-term efficacy and maximize patient selection and technique.

## CRediT authorship contribution statement

**Fathi Milhem:** Conceptualization, Methodology, Investigation, Data curation, Formal analysis, Writing – original draft, Writing – review & editing, Visualization. **Muhammad Takhman:** Conceptualization, Methodology, Investigation, Data curation, Formal analysis, Writing – original draft, Writing – review & editing, Visualization. **Mohamed S. Elgendy:** Conceptualization, Methodology, Investigation, Data curation, Formal analysis, Writing – original draft, Writing – review & editing, Visualization. **Anas Abu Zahra:** Conceptualization, Methodology, Investigation, Data curation, Formal analysis, Writing – original draft, Writing – review & editing, Visualization. **Sarah Saife:** Conceptualization, Methodology, Investigation, Data curation, Formal analysis, Writing – original draft, Writing – review & editing, Visualization. **Sakeena Saife:** Conceptualization, Methodology, Investigation, Data curation, Formal analysis, Writing – original draft, Writing – review & editing, Visualization. **Waseem Shehadeh:** Conceptualization, Methodology, Investigation, Data curation, Formal analysis, Writing – original draft, Writing – review & editing, Visualization. **Mohammad Bdair:** Conceptualization, Methodology, Investigation, Data curation, Formal analysis, Writing – original draft, Writing – review & editing, Visualization. **Omar Abu-Khazneh:** Conceptualization, Methodology, Investigation, Data curation, Formal analysis, Writing – original draft, Writing – review & editing, Visualization. **Yazan Hamdan:** Conceptualization, Methodology, Investigation, Data curation, Formal analysis, Writing – original draft, Writing – review & editing, Visualization. **Qutayba Z. Ayaseh:** Conceptualization, Methodology, Investigation, Data curation, Formal analysis, Writing – original draft, Writing – review & editing, Visualization. **Orabi Hajjeh:** Conceptualization, Methodology, Investigation, Data curation, Formal analysis, Writing – original draft, Writing – review & editing, Visualization. **Ayesha Younas:** Conceptualization, Methodology, Investigation, Data curation, Formal analysis, Writing – original draft, Writing – review & editing, Visualization. **Walaa Abu Alya:** Writing – review & editing, Visualization. **Ahmad Mohammad:** Conceptualization, Methodology, Investigation, Data curation, Formal analysis, Writing – original draft, Writing – review & editing, Visualization. **Anwar Zaitoun:** Conceptualization, Methodology, Investigation, Data curation, Formal analysis, Writing – original draft, Writing – review & editing, Visualization.

## Ethics approval and consent to participate

Not applicable. This study is a systematic review of previously published sham-controlled trials; it did not involve any new data collection from human participants or animals, and therefore did not require ethics committee approval or participant consent.

## Consent for publication

Not applicable. This manuscript does not contain any individual-level participant data or identifying information requiring consent for publication.

## Availability of data and materials

All data analyzed in this systematic review were obtained from publicly available sources (the cited literature). No new datasets were generated. The data supporting the conclusions are included in the referenced studies; additional information can be provided by the corresponding author upon reasonable request.

## Funding

This research received no external funding.

## Conflict of interest statement

The authors declare that they have no conflicts of interest relevant to the content of this article. No financial or personal relationships influenced the conduct or reporting of this research.

## List of Abbreviations

**AE**: Adverse Event
**BMI**: Body Mass Index
**CI**: Confidence Interval
**GAE**: Genicular Artery Embolization
**IQR**: Interquartile Range
**ITT**: Intention-to-Treat
**KOA**: Knee Osteoarthritis
**KOOS**: Knee Injury and Osteoarthritis Outcome Score
**MCID**: Minimal Clinically Important Difference
**MD**: Mean Difference
**NNT**: Number Needed to Treat
**NSAID**: Nonsteroidal Anti-Inflammatory Drug
**OR**: Odds Ratio
**PRISMA**: Preferred Reporting Items for Systematic Reviews and Meta-Analyses
**PROSPERO**: International Prospective Register of Systematic Reviews
**QoL**: Quality of Life
**RCT**: Randomized Controlled Trial
**RR**: Relative Risk (Risk Ratio)
**SAE**: Serious Adverse Event
**SD**: Standard Deviation
**SMD**: Standardized Mean Difference
**VAS**: Visual Analogue Scale
**WMD**: Weighted Mean Difference
**WOMAC**: Western Ontario and McMaster Universities Osteoarthritis Index
